# Motor Cortex Stimulation in Parkinson's Disease

**DOI:** 10.1155/2012/502096

**Published:** 2012-11-08

**Authors:** Marisa De Rose, Giusy Guzzi, Domenico Bosco, Mary Romano, Serena Marianna Lavano, Massimiliano Plastino, Giorgio Volpentesta, Rosa Marotta, Angelo Lavano

**Affiliations:** ^1^Department of Neurosurgery, University Hospital of Germaneto, Campus “Salvatore Venuta”, Viale Europa, 88100 Catanzaro, Italy; ^2^Department of Neurology, General Hospital, Crotone, Italy; ^3^Department of Psychiatry, University Hospital of Mater Domini, Catanzaro, Italy

## Abstract

Motor Cortex Stimulation (MCS) is less efficacious than Deep Brain Stimulation (DBS) in Parkinson's disease. However, it might be proposed to patients excluded from DBS or unresponsive to DBS. Ten patients with advanced PD underwent unilateral MCS contralaterally to the worst clinical side. A plate electrode was positioned over the motor cortex in the epidural space through single burr hole after identification of the area with neuronavigation and neurophysiological tests. Clinical assessment was performed by total UPDRS, UPDRS III total, UPDRS III-items 27–31, UPDRS IV, and UPDRS II before implantation in off-medication and on-medication states and after surgery at 1, 3, 6, 12, 18, 24, and 36 months in on-medication/on-stimulation and off-medication/on-stimulation states. We assessed changes of quality of life, throughout the Parkinson's disease quality of life scale (PDQoL-39), and the dose of anti-Parkinson's disease medications, throughout the Ldopa equivalent daily dose (LEDD). During off-medication state, we observed moderate and transitory reduction of total UPDRS and UPDRS total scores and significant and long-lasting improvement in UPDRS III items 27–31 score for axial symptoms. There was marked reduction of UPDRS IV score and LEDD. PDQL-39 improvement was also significant. No important complications and adverse events occurred.

## 1. Introduction

Deep brain stimulation (DBS) represents the gold standard for surgical treatment in patients with Parkinson's disease (PD), but unfortunately it is not fully effective in controlling each motor sign, and adverse effects are common. However, DBS cannot be always proposed to all PD patients because very often do not fill into the inclusion criteria for this procedure. Recently, other minimal invasive neuromodulation procedures with low morbidity-mortality and more suitable for cases excluded from DBS or unresponsive to DBS could be considered. Among these, motor cortex stimulation (MCS) may be one of the new opportunities [1–4] first introduced by Canavero back in 2000. In 2003, Pagni et al. spearheaded an Italian Multicenter Study on 41 PD patients treated with extradural MCS and long-term results have been reported in 2008 [5]. He showed that any symptom was modulated by MCS without a clear predictability. Thereafter, Pagni found a statistically significant improvement on the UDPRS III at 1, 3, and 6 months with a trend back to baseline, thereafter, and L-dopa-induced dyskinesias; painful dystonia and motor fluctuations were satisfactorily controlled. Other small case series of PD patients treated with extradural MCS (EMCS) have been reported with variable clinical results [6–9]. Anyway all these previous studies were open-label since in 2011, Moro et al. reported the double-blinded outcomes from unilateral subdural MCS [10].

The aim of this prospective observational study was to investigate the efficacy and the safety of unilateral extradural MCS in a select group of severe affected PD patients in which DBS was not indicated or refused.

## 2. Materials and Methods

Ten patients affected by primary advanced Parkinson's disease (6 men and 4 women; mean age 71 years; range 56 to 83 years old) were enrolled and underwent the unilateral implant of a epidural plate electrode over the motor cortex between April 2006 and April 2009 at the Department of Neurosurgery of the University Hospital of Catanzaro.

The inclusion criteria were idiopathic PD with at least 5 years duration, total UPDRS in off condition ≥40/180, Hoehn and Yahr stage ≥3, severe motor fluctuations plus disabling dyskinesias, UPDRS improvement to L-dopa challenge test ≥30%, DBS not indicated or refused, and lack of eligibility for DBS (i.e., refused by the patient or contra-indicated according to the Core Assessment Program for Surgical Interventional Therapies in PD (CAPSIT-PD) [11] with the only exception of the age criterion >70 years).

The exclusion criteria were history of epileptic seizures, evidence of major psychiatric issue (except antiparkinsonian drug induced), significant or unstable medical disorders (coagulopathies, serious heart or pulmonary disease, uncontrolled hypertension, or diabetes), alcohol and drug abuse, severe cognitive deterioration, and previous neurosurgical treatments.

Surgery was performed under local anaesthesia with a mild IV sedation if required. We used craniometer landmarks (10–20 EEG system) and Taylor-Hanghton lines to draw the central sulcus over the scalp. Primary motor cortex (M1) was identified with high resolution CT scan, MRI with fiducial markers and neuronavigation. A single burr hole was made in front of the central sulcus, and a quadripolar plate electrode (Resume, Medtronic, Inc.) was slipped epidurally over the motor strip at the hand knob. In all patients, the electrode implant was monolateral and performed controlaterally to the most affected side. The correct position of the electrode was verified neurophysiologically using somatosensory evoked potentials (SSEPs) to identify the central sulcus, and motor evoked potential (MEP) to identify the primary motor cortex (M1). For SSEPs, the “N20/P20 wave phase reversal technique” was used after stimulation of the controlateral *median* nerve at the wrist using a 20 mA–100 microsec. monopolar square pulse at a rate of 4.32 HZ; SSEPs were recorded from the Resume electrode in monopolar montage (all referenced to the 10–20 location of Fpz). A cortical N20 potential was recorded over the sensory cortex, and a cortical P20 potential was recorded over the motor cortex; the central sulcus was located between the two contacts showing the phase reversal.

The MEP was obtained by motor cortex focal anodal stimulation through two adjacent contacts of the same plate electrode with short train of stimuli (5 stimuli each, rate of 5 trains per second, 500 microsec. pulse, 4 millisec. interspike interval). Muscle responses were recorded with EMG needles from biceps brachii, abductor pollicis brevis, and quadriceps of the opposite hemibody.

After the neurophysiologic tests, the plate electrode was placed over the long axis of the motor cortex and externalized with a percutaneous connection behind the ear. An external stimulation period of 2-3 weeks was performed for detection of the most beneficial parameters of stimulation and the adverse effects. Starting from 24 hours after electrode implantation, we checked all contacts in a bipolar setting using low frequencies (20–40 Hz) and high pulse widths (180–210 microsec): the amplitude was slowly raised until the appearance of adverse motor movements and sensory phenomenons, and then it was decreased to the movement and sensation subthreshold voltage (2.5–4.0 V). Unified Parkinson's disease rating scale for motor examination (UPDRS III total) was used to score the initial benefits of stimulation. The effect of each setting was assessed after 60 min of continuous on-stimulation and after a subsequent washout period of 30 min off-stimulation.

The epidural electrode was then connected to a pulse generator (Soletra or Kinetra, Medtronic, Inc.) implanted in a subclavicular subcutaneous pocket. Chronic stimulation began with the most efficacious setting obtained during test period (2.5–4.0 V, 40 Hz, and 180 microsec), continuously delivered night and day through the most distant contacts of the plate electrode under a bipolar configuration.

Patients were assessed preoperatively (baseline time) and after 1, 3, 6, and 12 months and at least every 6 months after surgery with both stimulator on (on-Stim) and 2 weeks after switch off (off-Stim) by a neurologist who was blinded to the condition of the patient. The assessment was performed with total UPDRS, UPDRS III total, UPDRS III items 27–31, UPDRS IV, and UPDRS II. Select items of the UPDRS III such as limb tremor, rigidity, and bradykinesia were evaluated controlateral and ipsilateral. The evaluations were carried out 12 hours after medication withdrawal (off medication) and 90 minutes after the first levodopa dose (on medication). Anti-Parkinson's disease medications were not changed during the month before surgery and up to three months after surgery. Changes in the settings of stimulation were done from the 3 month followup and were directed by the clinical assessments at each followup visit; they included increase in amplitude and change in frequency. Effective and final stimulation parameters were 3.5–4.7 V, 40–80 Hz, and 180 microsec., continuously delivered through contacts 0−/3+.

Quality of Life was assessed with the Parkinson's disease Quality of life scale (PDQoL-39) preoperatively and 6, 12, 18, and 24 months after surgery.

Dose of anti-Parkinson's disease medications (L-dopa and dopamine agonists) was evaluated with L-dopa equivalent daily dose (LEDD) at 6 12, 18, and 36 months. Movie recordings for motor assessment before and after stimulation were performed.

Patients were followedup for 36 months: eight patients brought the 36 months followup, while two patients died after the 24 months of followup. There were two substitutions of expired IPG, respectively, 30 months and 32 months after the implant.

## 3. Statistical Analysis

Data were expressed as means ± SD. An ANOVA test for independent samples was performed to compare the means. A *χ* ^2^ test was performed to compare prevalences. In all cases, a *P*-value of 0.05 was considered statistically significant. All comparisons were performed using the statistical package SPSS 17.0 for Windows (SPSS, Chicago, IL, USA).

## 4. Results

**Figure 1:**
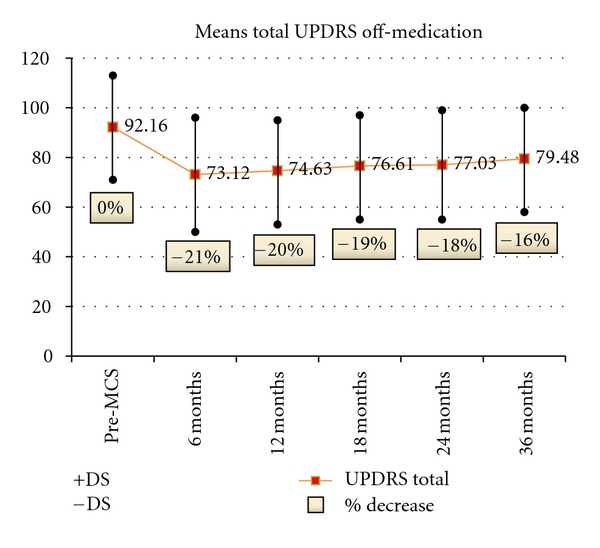


**Figure 2:**
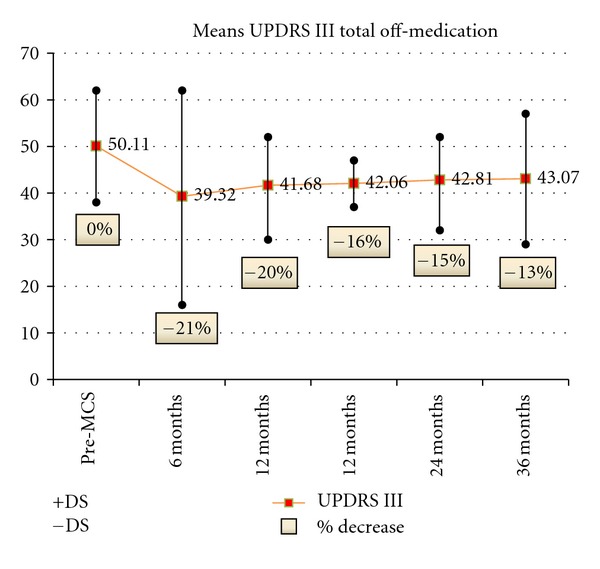


Moderate improvement of both total UPDRS and UPDRS III total in off-medication condition was observed in all patients, decreasing after 12 months in spite of changes in stimulation parameters and stimulation mode. Nevertheless, total UPDRS and UPDRS III total scores at 24 and 36 months were always lower than at preoperative evaluation (Figures 1 and 2). Improvement was only very moderate in the on-medication state.

**Figure 3:**
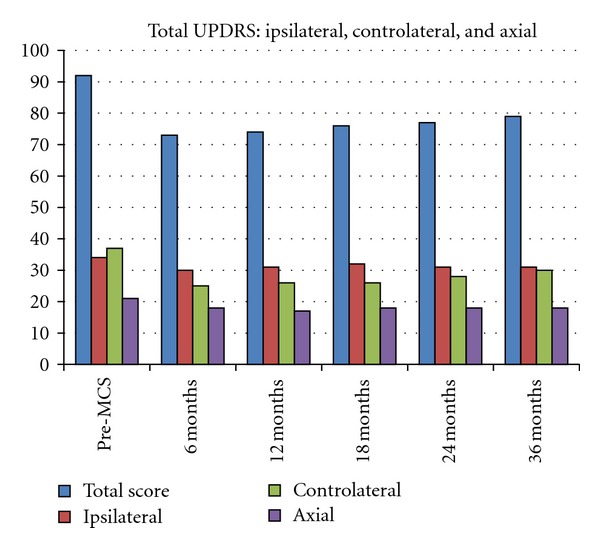


The benefits on limbs tremor, rigidity, and bradykinesia were simultaneous and bilateral, slight evident in the hemibody opposite to the stimulated side (Figure 3).

**Figure 4:**
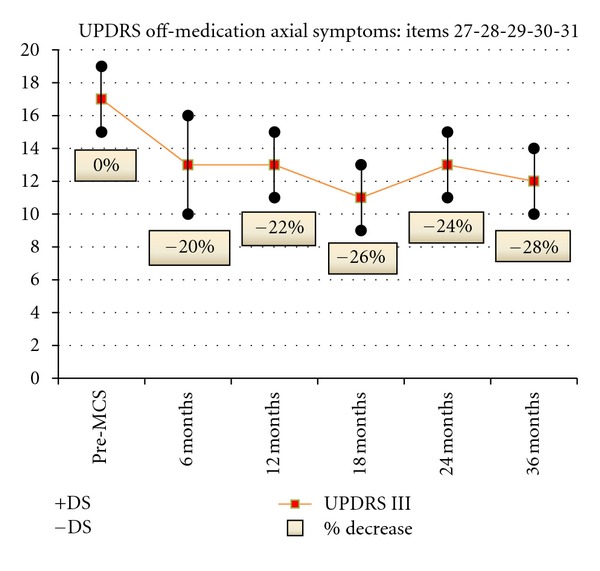


Greater significant and long-lasting improvement was observed in axial symptoms as measured by the UPDRS III items 27–31 off medication (mean percentage of decrease: 25% at 1 month, 30% at 3 months, 20% at 6 months, 22% at 12 months, 26% at 18 months, 24% al 24 months, and 28% at 36 months) (Figure 4).

Although the improvement of UPDRS III total score in off medication was low, the clinical benefits of those items had important impact on both patient self-grooming and psychology and on the assistance needed. There was a subgroup of patients severely handicapped owing to difficult in standing and deambulation which were very significantly improved.

**Figure 5:**
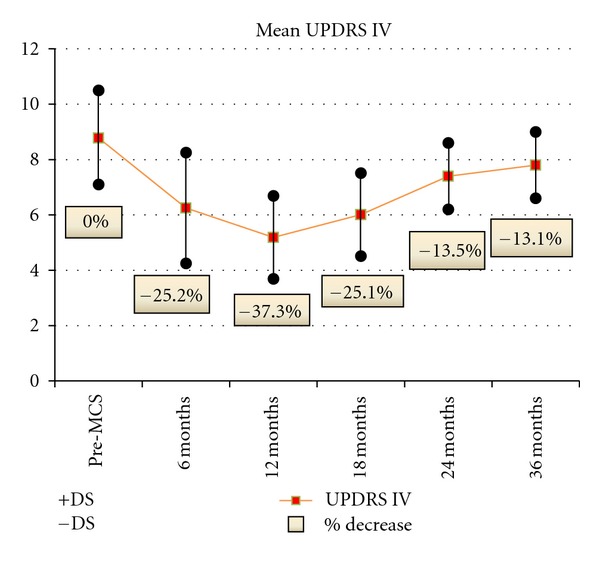


There was marked attenuation of L-dopa-induced dyskinesias and dystonia with significant reduction of UPDRS IV score up to 18 months (mean percentage of decrease: 29.6% at 6 months, 40.9% at 12 months, 31.8% at 18 months, 15.9% at 24 months, and 11.4% at 36 months) (Figure 5). 

Eight patients reported reduced off time in clinical fluctuations (UPDRS IV item 39 score from 3 to 1).

**Figure 6:**
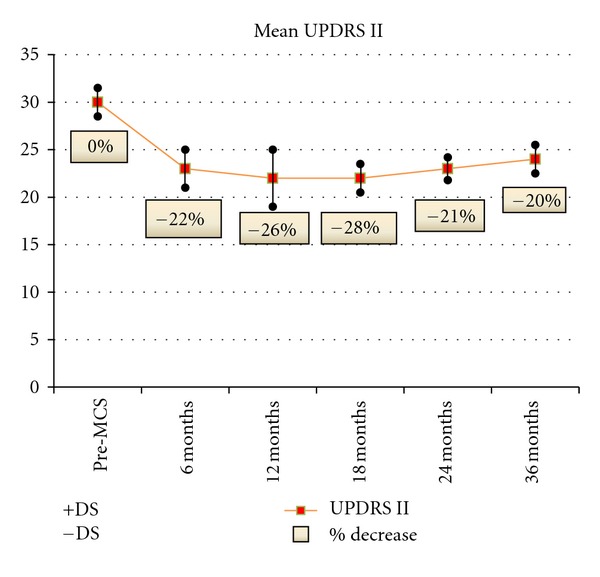


Mean UPDRS II score showed significant and prolonged amelioration of activities of daily living with most pronounced clinical benefits in speech, freezing and walking (Figure 6).

**Figure 7:**
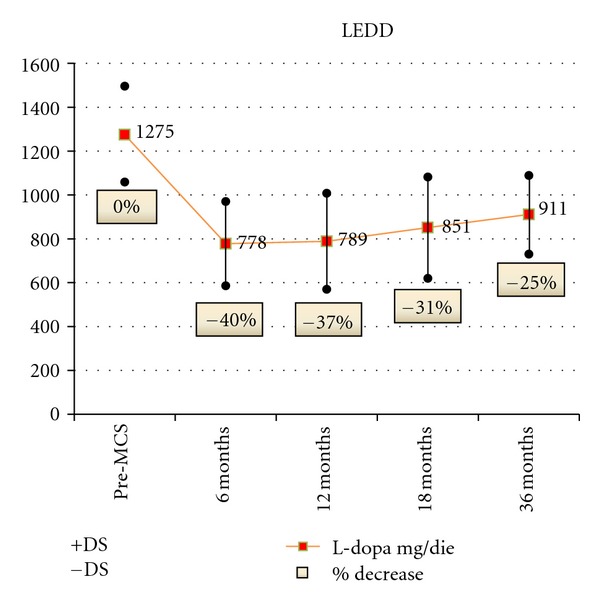


Also LEDD documented evident reduction of L-dopa and dopamine agonists dosage (mean percentage of decrease: 39% at 6 months, 38% at 12 months, 33% at 18 months, 37% at 24 months, and 29% at 36 months) (Figure 7).

**Figure 8:**
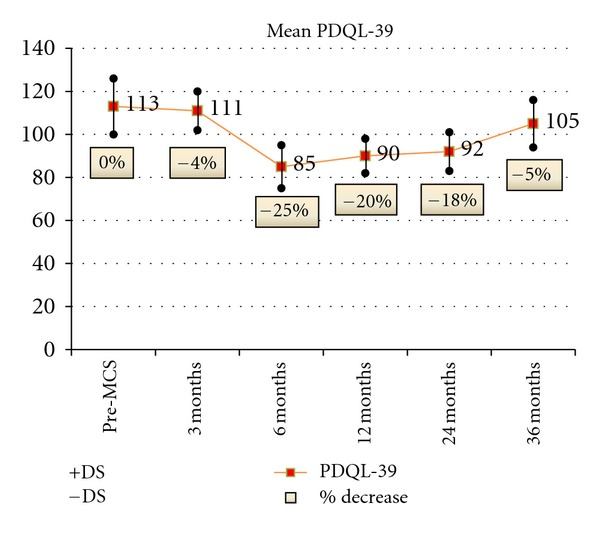


Concerning PDQL-39, the improvement was significant from 6 months to 24 months evaluation (mean percentage: 25% at 6 months, 20% at 12 months, and 18% at 24 months) with a following trend to return nearly to the baseline value (Figure 8).

In four patients, there was early benefit while in six patients there was delay of 1-2 weeks between the starting of stimulation and full evidence of symptoms improvement.

No complications and no adverse events occurred except for the appearance of local pain on the implant site of the plate electrode described in three patients when switching the stimulation on; the bipolar coagulation of the dura around the electrode relieved this complication.

In two patients, exhaustion of IPG occurred with worsening of symptoms within three and five days, respectively, afterwards the IPG substitution both patients improved over previous baseline.

## 5. Discussion

Chronic MCS has been used not only in relieving refractory pain but also in improving a variety of movement disorders including PD, tremor, and poststroke dystonia [2, 4, 5, 9, 12–17]. Single case reports, multicenter retrospective clinical review and small case series of advanced PD patients treated with EMCS have been reported with variable clinical results [1–4, 6–9, 16]. There are only three prospective case series [6, 8, 9]. Arle ana Shils [9] reported a significant effect on overall motor performance as assessed by UPDRS while Cilia et al. [8] found that extradural MCS produced no motor benefit but subjective improvement involving mainly axial symptoms as well as reduction in daily off time and dyskinesias. Gutiérrez et al. [6] confirmed the absence of significant modification of UPDRS III scores with only mild daily life activities improvement and slightly reduction of LEDD. The methodology of these prospective studies was fairly consistent with a evident degree of intra-cohort variability. Anyway, all these previous studies were open label. In 2011, Moro et al. [10] reported the double-blinded outcomes at 3 months and unblinded outcomes at 1 year from unilateral subdural MCS in five patients with advanced PD. The authors concluded that no significant clinical benefits were evident on parkinsonian symptoms in both outcomes, but five patients represent a very restricted sample for drawing significant conclusions. Nevertheless, some items of the UPDRS, such as gait and balance, during off medication showed a slight improvement in on-MCS in Moro's PD patients (−17,4% and −5,3%, resp.).

Our study is a single-center prospective observational study, and the results suggest that extradural MCS can modulate the cardinal symptoms in advanced PD patients. The improvement concerned mainly axial symptoms, L-dopa-induced dyskinesias and dystonia, quality of life, and global condition (Table 1). The benefit on limbs tremor, rigidity and bradykinesia were bilateral but not significant, with a slight prevalence in the hemibody opposite to the stimulated side. EMCS proved to be safe as no complications or adverse effects were reported.

Many previous data highlighted the strong involvement of motor cortex in PD [3]. Functional neuroimaging studies showed that primary motor cortex (M1) is hypohyperactive in both early and late stages of PD. In patients with early untreated PD, fMRI showed M1 hypoactivation [18]; conversely in advanced parkinsonism, M1 and lateral premotor cortex (L-PMC) were found to be hyperactive [19]. M1 hyperactivity has been attributed to cortical reorganization resulting from drug-induced reafferentation of the deficient subcortical motor system [20].

Increasing of the corticospinal projections at rest resulting from a reduced intracortical inhibition could be implicated in rigidity [21], while intracortical or thalamocortical facilitatory inputs may not correctly activate all the cortical area involved in movement preparation and execution leading to bradykinesia [22].

Motor cortex can be stimulated either not invasively using transcranial magnetic stimulation (TMS) or invasively using surgically positioned stimulating plate electrode like in extradural or subdural cortical stimulation (MCS).

It has been showed that both repetitive TMS and MCS improve motor performances in PD [23–25].

The motor cortex region is the final common link between deeper circuitry coordinating movement and the spinal cord itself. It is one of the few areas in which the pyramidal and extrapyramidal systems interact. Movement disorders may therefore, respond to some type of stimulation of cells in this region. The motor cortex is connected to the basal ganglia indirectly via a corticostriatal pathway and directly via a corticosubthalamic circuit. MCS may exert its effect modulating the subthalamic nucleus (STN) directly or through the loop cortex-striatum-lateral globus pallidus-STN [6]. Chronic MCS may alter not only the firing patterns in the basal ganglia, but also, due to its location, the interactions between the pyramidal and extrapyramidal systems [9]. MCS may also modulate the activity of supplementary motor area (SMA) or modulate the “suppressor cortical system” [5].

The topography and extension of the somatotopic representations within the motor cortex showed modification in advanced PD with progressive enlargement and displacement of hand motor map; this can explain the improvement of axial symptoms with plate electrode implanted over the motor strip at the hand knob [5].

Bilateral effects of unilateral MCS are due to bi-directional interconnectivity between motor cortex and other neural structures located in the cortex, basal ganglia, and thalamus. Transcallosal pathways are the most responsible for these bilateral effects; interhemispheric conduction pathways exist between the hand representations of motor cortex but also weaker transcallosal connection for body parts exists outside hand areas which explains why the hand area should be targeted for MCS in PD [26].

The clinical changes induced by MCS are usually delayed; time consuming process of synaptic plasticity, long-term potentiation or depression, expression of secondary messengers, or polarization of brain tissue may consequently also hypothesised as possible reasons for this delay [1, 5, 12, 27, 28]. Also the observed benefit persisting for some days after IPG failures might be due to a plastic modification of the central neural circuits.

The choice of stimulation parameters is made on an empirical basis. The amplitude of stimulation is always subthreshold for movements and sensations, while both low and high frequency are used with positive results [5, 6, 9, 25, 29]. We utilized low frequency stimulation (40–60 Hz) with 180 microsec pulse width according with Canavero's works [12, 25].

Long-term L-dopa syndrome symptoms, dyskinesia, and dystonia are significantly reduced in most of reported patients treated with MCS; the explanation might be that the doses of dopaminergic drugs are reduced besides a direct effect of cortical stimulation on symptoms [13, 30].

Clinical effects of MCS cannot be compared with STN DBS because of the different inclusion criteria and the small number of patients treated by MCS. However, STN DBS appears to be more effective on motor symptoms while MCS on axial symptoms [4, 5, 16, 28, 30, 31].

Complication rate and adverse events are low for extradural MCS [31–33]. Occurrence of sporadic epileptic seizures has been reported during test stimulation in a minority of patients while epilepsy evoked by chronic MCS has not been reported. Indeed, stimulation parameters used in human series are either clinically or electroencephalographically epileptogenic [32]. The most serious complication is epidural hematoma which is very rare making the risk of perioperative hemorrhage much lower compared to DBS in which most reported series suggest a intracerebral hemorrhagic risk <2%, with a range of 0.2% to 9.5% [34, 35]. Local pain in the site of plate electrode implant is also reported during the stimulation; it may be relieved by superficial denervation of the dura performed with its incision and resuturing around the electrode or with bipolar coagulation on its surface [33].

Several authors suggested that MCS has a long standing effectiveness [5, 30], while other authors reported short lasting benefits within the first 12 months [9]. In our patients, there was a reduction of effects after 12 months of stimulation documented by total UPDRS and UPDRS III total evaluations, despite increases of stimulation amplitude and changes in the other stimulation parameters. This reduction may be due to habituation of the cortex to stimulation, but also the progression of PD may be considered. Conversely benefits on axial symptoms, L-dopa induced dyskinesias, and activities of daily living were more long-lasting.

Our study have some limitation. First, evaluations were conducted in an open-label fashion, and this does not allow to exclude a placebo effect. Furthermore, assessment in off-stimulation condition performed 2 weeks after IPG switch-off may be too early to determine the true MCS effectiveness since the effect of stimulation could outlast 3-4 weeks. Finally, clinical features as axial symptoms and dyskinesias can be better evaluated using specific scales rather than UPDRS.

## 6. Conclusions

Based on our small sample studied, we can sustain that extradural MCS moderately improves all main symptoms in severe advanced PD patients with greater effectiveness on axial symptoms and complications of anti-Parkinson's disease medication treatment. Furthermore, anti-Parkinson's disease drug intake is reduced. Our results are less evident than DBS, but EMCS has some advantages; firstly it can be performed without use of frame-based stereotactic techniques and secondly it does not require intracerebral introduction of micro or macroelectrodes with consequent lower risk of hemorrhage. Moreover, unilateral extradural MCS is effective bilaterally with reduction of the procedure costs if compared with DBS.

Although these positive findings of large studies with double-blinded evaluation of clinical results are needed to better determine if extradural, MCS should be considered as surgical option in advanced Parkinson's disease with predominant axial symptoms, gait disturbances, and therapy complication when other treatments have failed.

## Figures and Tables

**Table 1:** Changes of quality of clinical assessment in ten patients with advanced Parkinson's disease performed before motor cortex stimulation and after surgery at 6, 12, 24, and 36 months in off-medication/on-stimulation states.

	Pre-MCS	6-Months	12-Months	24-Months	36-Months
UPDRS total	92,2 ± 21,7	73,1 ± 23,3*	74,6 ± 21,4*	77 ± 22,6*	79,5 ± 21,2^§^
UPDRS II	30,1 ± 1,5	23,5 ± 1,5	22,3 ± 3	23,8 ± 1,4	24,1 ± 1,5
UPDRS III	50,1 ± 12,7	39,3 ± 23,3^§^	41,7 ± 11,4^§^	42,8 ± 9,3^§^	43,1 ± 14,3^§^
UPDRS IV	8,8 ± 1,7	6,2 ± 1,8	5,2 ± 2^§^	7,4 ± 1,2	7,8 ± 1,2
UPDRS III axial symptoms: items 27–31	17 ± 2,2	13,6 ± 2,7*	13,3 ± 2,9*	12,9 ± 2,1*	12,2 ± 1,9*
LEDD (mg/die)	1275 ± 216	778 ± 192^§^	789 ± 219^§^	810 ± 180^§^	911 ± 178^§^
PDQoL-39	113,5 ± 13,3	85,1 ± 9,3^§^	90,8 ± 10,2^§^	93,1 ± 8,9^§^	107,8 ± 11,3

MCS: motor cortex stimulation; UPDRS: unified Parkinson disease rating scale; LEDD: L-dopa equivalent daily Dose; PDQoL-39: Parkinson's disease quality of Life scale. *P *values calculated versus pre-MCS. *P = .001; ^§^ *P* = .005.
